# The Effects of Pandemic Restrictions on Public Health—Improvements in Urban Air Quality

**DOI:** 10.3390/ijerph19159022

**Published:** 2022-07-25

**Authors:** Gabriela Cioca, Raluca Andreea Nerişanu

**Affiliations:** 1 Preclinical Department, Faculty of Medicine, Lucian Blaga University of Sibiu, 550024 Sibiu, Romania; gabriela.cioca@ulbsibiu.ro; 2 Department of Finance and Accounting, Faculty of Economic Sciences, Lucian Blaga University of Sibiu, 550324 Sibiu, Romania

**Keywords:** NO_2_, SO_2_, CO, PM, air pollution, pandemic, COVID-19, air quality

## Abstract

The present study aims to provide evidence on the effects of pandemic curtailment measures on public health, targeting the changes in breathable air quality, within urban areas. The analyzed period covers the full impact of lockdowns in Europe in 2020. We used everyday data for each analyzed pollutant, NO_2_, SO_2,_ CO, PM_2.5_ and PM_10_, from urban monitoring stations that provided real-time concentrations (provided by Copernicus Atmosphere Monitoring Service, Environmental Protection Agency repository and European Environment Agency map services) and satellite data (provided by NASA Orbiting Carbon Observatory 2). In the present study, the urban air quality was computed using a composite index that was further analyzed in comparison with pandemic restrictions. Descriptive statistics, charts and maps were used to visualize the data that covered the analyzed countries. Our results show that air pollution was reduced by 12% after lockdowns in European urban areas, with a 0.76 correlation between air pollution and pandemic restrictions. All air pollutants registered significant drops.

## 1. Introduction

As an adult inhales approximative 11,000 L/day of air [1], it is highly important that the quality of the air is reasonably high and consists of a normal state of the usual components of terrestrial atmosphere (nitrogen, oxygen and a tiny percent of argon, carbon dioxide, neon, helium and hydrogen) [2]. Anthropogenic or natural events can adjust these concentrations or add new components to the air structure, such as carbon dioxide (CO_2_), carbon monoxide (CO), nitrogen dioxide (NO_2_), particle matter (PM), volatile organic compounds (VOCs), ozone (O_3_), sulfur dioxide (SO_2_), methane (CH₄) or other gases that can have a negative impact on human or environmental health [3,4].

The COVID-19 lockdown period imposed severe restrictions regarding industrial activity and human mobility; thus, it is the best opportunity to test if the reduction of these types of activities had a significant impact over the quality of air, thus improving the public health in urban areas.

The severe acute respiratory syndrome coronavirus-2 (SARS-CoV-2) first emerged in China in December 2019, but has spread rapidly since, quickly becoming a global problem, being declared a pandemic by the World Health Organization (WHO) on 11 March 2020. The governments of the affected countries have imposed many restrictions to human interactions in order to stop the virus transmission. Symptoms are extremely heterogenous, although the virus primarily affects the respiratory system [5]. COVID-19 is primarily transmitted through physical contact or oral transmission [6,7]. Other studies reported surface contamination up to 72 h on plastic materials [8]. A study showed that protection is increased when physical distancing exceeds 1 m and eye and face protection are worn, with a significant difference for N95 or similar respiratory masks in comparison with normal chirurgical masks [9]. Thus, in order to diminish the virus transmission of SARS-CoV-2, some human mobility restrictions and control measures were regulated, first in China [10] and later in Europe and the rest of the world.

Breakdown hit Europe in March 2020, starting from Italy, when the first cases of COVID-19 appeared [11], resulting in fast restrictions from government, starting from 15 March and consisting of human mobility, travel and activity restrictions, along with behavioral practices enforced by law, such as mask wearing and social distancing, as they were the immediate protection against the virus transmission [9,12]. Initial restrictions were more severe than the ones prolonged across the whole 2020 year. These restrictions were put in place sequentially in Europe, as the situation evolved in each country.

Within 2020, energy demand dropped by 25 percent every week on average when full lockdown was imposed and by 18 percent on average when partial lockdown was imposed in comparison with the normal energy demand [13]. Total energy demand dropped by an average of 3.8% in the first quarter of 2020 compared to the first quarter of 2019 according to global measurements [13]. The biggest impact in Europe was registered in March, when confinement measures were the most substantial. Coal demand dropped by 8% from the first quarter of 2020 in comparison with that reported in the same period of 2019, while oil demand dropped by 5%, especially because of curtailment measures in mobility and aviation (which account for almost 60% of the oil demand) [13]. The same source specified that diesel consumption dropped by 1.5 million barrels per day and gasoline consumption by 1.7 million barrels per day, from 2019 to 2020, for the first quarter of the year [13]. Gas demand dropped by 2% from the first quarter of 2019 to the first quarter of 2020.

As most transportation and industrial activities were restricted and fossil fuel combustion is a primary source for most of the air pollutants which are found to be responsible for human health damage, the hypothesis that the restrictions have lowered the air pollutant concentrations from the ground-level layer will be argued in the present paper.

## 2. Materials and Methods

### 2.1. Data Source

The present study provides data on 28 European countries over a period of 10 months. The countries were selected from the European continent, taking into consideration the data availability level for each state, thus selecting the ones which had over 70% data availability. Data on air pollutants were extracted from urban monitoring stations implemented in Europe, measured as real-time concentrations from the Environmental Protection Agency repository (link available in the data availability statement). Also, satellite data were obtained by accessing the NASA Orbiting Carbon Observatory 2 product, through the Copernicus Atmosphere Monitoring Service. Furthermore, meteorologic conditions such as wind, temperature or precipitation were obtained from the ERA5 satellite through the Copernicus Atmosphere Monitoring Service (link also available in the data availability statement).

Restrictions in the COVID-19 pandemic were obtained from the European Centre for Disease Prevention and Control repository, for each day starting from 15 of March 2020, while data on the impact of NACE rev. 2 activities on air quality and the impact of the main activities of manufacturing and industry over the national value added were obtained from Eurostat.

Air quality missing data were linearly extrapolated using previous values. Data completeness statistics were concluded at 96% (4% of the data was estimated).

Table 1 presents the sources for the data integrated in the present analysis.

### 2.2. Air Pollution Index (API) and Air Quality Index (AQI)

All data points regarding pollutants included in the air quality index were obtained in the US EPA standard, measured as the daily median for each country considered. Air quality data were normalized by applying the US EPA air quality index methodology, in order to homogenize all the analyzed pollutants and compute the air quality index for each country and for the entire area. Thus, for obtaining the air pollution score for each pollutant, Equation (1) was applied:

(1) $$I_{P}=\frac{I_{\mathrm{Hi}}-I_{\mathrm{Lo}}}{{\mathrm{BP}}_{\mathrm{Hi}}-{\mathrm{BP}}_{\mathrm{Lo}}}\left(C_{P}-{\mathrm{BP}}_{\mathrm{Lo}}\right)+I_{\mathrm{Lo}};$$

where I_P_ is the air pollution score for each pollutant, C_P_ is the period average truncated concentration for each pollutant (as PM_2.5_ and CO have to be truncated to 1 decimal place, and PM_10_, SO_2_ and NO_2_ have to be truncated to integer), BP_Hi_ is the concentration breakpoint first to exceed the C_P_, BP_Lo_ is the concentration breakpoint first below the C_P_, and I_Hi_ and I_Lo_ are the AQI values corresponding to BP_Hi_ and BP_Lo_, respectively. The breakpoints for each pollutant and the corresponding AQI values are available in the US EPA air quality index methodology (link available in the data availability statement). After calculating the I_P_, the score was normalized using linear normalization.

An air pollution index for each country and for each period was created using the average of the air pollution score; the index used PM, SO_2_, NO_2_ and CO data. Equal weights were attributed in order to compute the air pollution index.

The air pollution index (API) was composed as follows:

API (X) = avg [I_p_]; (2)

where X represents each country taken in consideration and I_P_ is the air pollution score for each pollutant considered.

An air quality index was created using the reciprocal of the air pollution index. The air quality index (AQI) was composed as follows:

(3) $$\mathrm{AQI}\;(X)=\frac{1}{\mathrm{API}\;\left(X\right)}$$

The meteorology data include total precipitation (m), near surface wind speed (m s^−1^), near surface air temperature (°K) and boundary layer height (m), which were obtained from the Copernicus Climate database using ERA5 analysis of meteorological data.

### 2.3. Restriction Index (RES)

The pandemic restriction data refer to the collection of data representing each restriction type that occurred each day in a specific country and that was aggregated using specific weights. In order to scale the weights with actual emissions data, we used the air emissions accounts by NACE Rev. 2 activity from the Eurostat database [14]. For example, the highest sulfur oxides (SO_2_ equivalent) emission is attributed, in this exact order, to transportation and storage, electricity, gas, steam and air conditioning supply and manufacturing. Transportation, storage, public administration, defense, and compulsory social security present the highest emissions of PM_2.5_, while agriculture, forestry, fishing, transportation, storage, manufacturing and construction are related to the highest emissions of PM_10._ The definitions for each activity contained in NACE Rev. 2 are available at the link presented in the data availability statement. Therefore, transportation contains activities related to air, water and land transportation, while storage reflects all the activities related to warehousing and support activities for transportation and postal and courier activities. As the repository does not allow us to break down activity classes into more specific activities and transportation and storage are the main activity sources for SO_2_ and PM_2.5_, as well as in the top five sources for PM_10_, we assume that air, water and land transportation are the main sources for these pollutants, while we do not have any evidence that a specific restriction affected the storage activity, other than the transportation process related to the storage activity. Thus, all restrictions related to transportation must have the maximum weight, although, in the EU, “road transport has the largest share of modes of transport” [15]. Similarly, regarding the main sources for PM_2.5_, the “public administration and defence; compulsory social security” class contains activities related to general public administration activities, foreign affairs, defense activities, justice and public order and compulsory social security activities. As the repository does not allow us to break down activity classes into more specific activities, we assume that all the restrictions related to the general public administration activities must have the maximum weight, while any restrictions related to defense or compulsory social security activities have not occurred. The class “electricity, gas, steam and air conditioning supply” covers activities related to electric power and gas generation, manufacture and distribution, and the production, collection and distribution of steam and air conditioning supply. Because the repository does not allow us to break down the activity class, we must assume that activities related to electric power and gas generation, manufacture and distribution, respectively, are the most pollutant activities, as there were not any restrictions to be found in relation to steam and air conditioning supply activities, and thus they do not affect the present research.

The maximum weight was attributed to: closing high schools, closing primary schools, closing sectors, stay home orders, teleworking, closing workplaces and closing daycares. Closing public activities, entertainment venues, hotels and other accommodations, restaurants and cafes, public transportation and restricting outdoor activities over 1000 were aggregated with a weight of 2, while closing gyms, sport centers and non-essential shops were aggregated with a minimum weight. In the aggregation process we used weights from 1 to 3.

A restriction index was created using summarization of the elevated restrictions in the selected periods, considering that the aggregation process of the mentioned restrictions was implemented for each day from the analyzed period.

The restriction index (RES) was composed as follows:

(4) RES (X) = sum_day1_ [restriction_1_ (X); restriction_2_ (X); … restriction_n_ (X)] + sum_day2_ [restriction_1_ (X); restriction_2_ (X); … restriction_n_ (X)] + … + sum_day14_ [restriction_1_ (X); restriction_2_ (X); … restriction_n_ (X)]

where X represents each country taken into consideration; restriction_1_ (X), restriction_2_ (X) and restriction_n_ (X) are the daily weights for each restriction of each country; and day 1, day 2… day 14 represent each day taken in consideration in each analyzed period.

### 2.4. Descriptive Statistics and Hypothesis Testing

As shown in Table 2, data were computed into bimonthly averages, considering that the period of incubation for COVID-19 symptoms could extend to 14 days [14], with a median of 5.2 days [5], and that governments usually took into consideration a two-week lockdown period. The ARX approach relates to the bimonthly periods. Moreover, visual maps were generated for each air pollutant, in three main periods: 1 March–30 May, 1 June–30 August and 1 September–30 October, in order to observe the seasonal changes. For the same reason, air pollution and restrictions over Europe and changes in air pollutant concentrations were constructed upon the same 3 periods: 1 March–30 May, 1 June–30 August and 1 September–30 October. Furthermore, the data used in the meteorological analysis are mapped for the three periods of 1 March–30 May, 1 June–30 August and 1 September–30 October.

Descriptive statistics refers to the central tendency indicators, coefficient of variation and correlation among the variables and also the relative modifications of each pollutant concentration, analyzed before and after the COVID-19 pandemic lockdown in Europe. Air pollution and restrictions were indexed for each analyzed period.

The research design is based on secondary data analysis of a time series.

To pursue our alternative hypothesis, that pandemic restrictions significantly affected air quality in urban areas, we ran a *t*-test with paired groups. For further forecasting, we used an ARX (autoregressive model with exogenous variable), enhancing a lag of 4 periods. We have also extended the regression to foster the best scenario.

**Figure 1:**
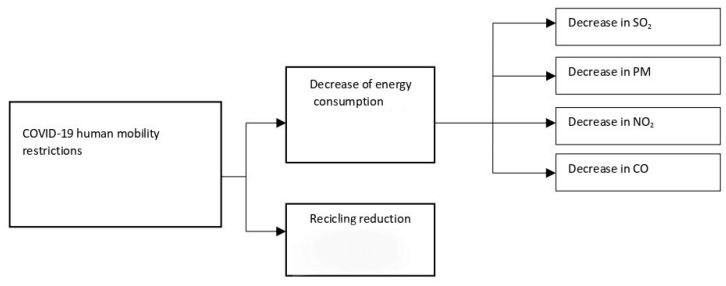
Curtailment restriction implications on public health [13].

Therefore, in order to summarize the curtailment restriction implications on public health, we have introduced a representative diagram as shown in Figure 1.

## 3. Results

### 3.1. Urban Air Pollution and Restriction Index Values

**Figure 2:**
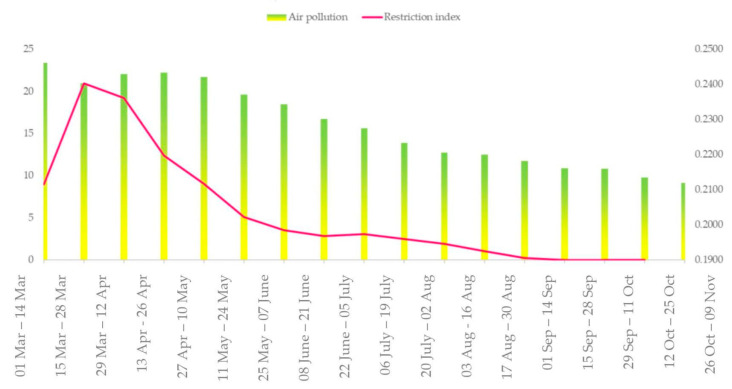
Air pollution (measured as air pollution index) and restrictions (measured as restriction index) during European lockdown and later.

Figure 2 shows the air pollution index (API) and the restriction index (RES) for each analyzed period, for the entirety of Europe, composed as follows:

API_UE_ = avg [API (X)]; (5)

RES_UE_ = avg [RES(X)]; (6)

where API_UE_ is the air pollution index for the entirety of Europe and RES_UE_ is the restriction index for Europe.

The European air pollution index is measured on a numerical scale from 0.19 to 0.25 units, where 0.25 is the maximum value and 0.19 is the minimum value, and the restriction index is measured in points, from 0 to 25 points, summarizing the restrictions for each period analyzed.

In Figure 2, the air pollution index, which is an aggregate index of NO_2_, CO, SO_2_, PM_10_ and PM_2.5_, had a decreasing pattern in urban areas as a result of energy reduction, traffic-related combustion reduction and industry breaks. Since energy dropped by 25% every week when full lockdown was active, and with an average decrease of 3.8% from spring 2019 to spring 2020 on the basis of the drop in traffic-related activities and partial or full industries breaks, air pollution settled on a decreasing trend, consisting of a total decrease of 12% in the analyzed period, which is the most significant air quality improvement since the industrial revolution [13]. As can be seen from Figure 2, air pollution in Europe hit the maximum in the first period, when the index registered was 0.246, and presented a decreasing pattern after the restrictions were put in place. The period with the highest restrictions was 28 March–10 April 2020, when the restriction index reached 22.3 points. Since lockdown, both the air pollution and restriction index had a decreasing trend, figuring the causal relationship of a self-determined model.

**Figure 3:**
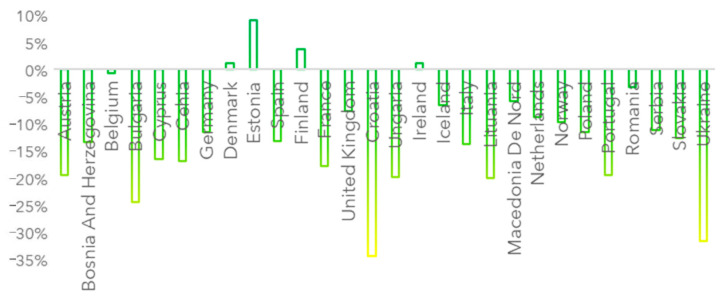
Air pollution changes (API_change_) from March to October 2020.

Figure 3 presents the main air pollution index changes (API_change_) from March to October, for each country considered, composed as follows:

(7) $${\mathrm{API}}_{\mathrm{change}}\;(X)=\frac{{\mathrm{API}}_{1}\;\left(X\right)}{{\mathrm{API}}_{0\;}\left(X\right)}\times 100-100\%$$

where ${\mathrm{API}}_{1}\;\left(X\right)$ is the air pollution index for a country X for the last period considered and ${\mathrm{API}}_{0}\;\left(X\right)$ is the air pollution index for a country X for the first period considered. The change refers to the last two weeks and the first two weeks of the analyzed period.

On average, pollution dropped by 12% from March to late October; the most positively affected countries were Ukraine (with a 27% drop in the air pollution index from 15 March to 25 October), Croatia (with a 29% drop from March to October), Bulgaria, Lithuania and Portugal.

When pollution is related to restrictions, the biggest impact was found in Croatia (1 unit of increase in restrictions generated a 0.50 decrease in pollution), followed by Bulgaria (0.25 decrease in air pollution for 1 unit increase in restriction index), Austria (0.21 decrease in air pollution for 1 unit increase in restriction index), Iceland and Lithuania (0.18 decrease, respectively, in air pollution for 1 unit increase in restriction index).

**Figure 4:**
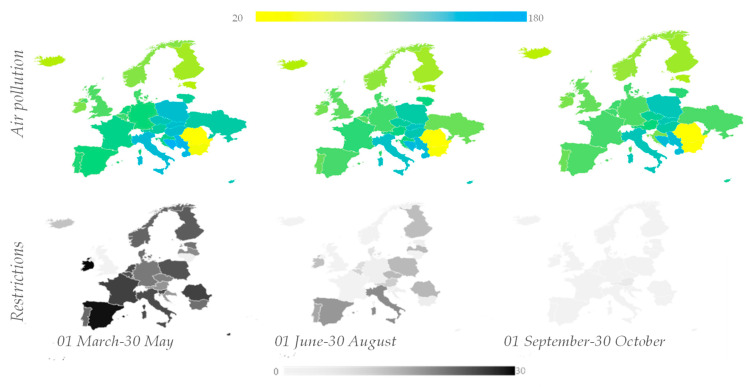
Urban air pollution (colored scale) and restrictions (black–grey scale) in Europe in three periods: 1 March–30 May, 1 June–30 August and 1 September–30 October.

Figure 4 presents the air pollution index (colored scale) and restriction index (black-grey scale) in each country analyzed, from the European Union, in three periods: 1 March–30 May, 1 June–30 August and 1 September–30 October, in order to observe the seasonal changes.

The values were composed as follows:

API_period y_ (X) = avg [API_X_]; (8)

where API_period y_ (X) represents the air pollution index for each of the three periods plotted (1 March–30 May, 1 June–30 August and 1 September–30 October) and X represents each country taken into consideration. All data points regarding pollutants were obtained in the US EPA standard, measured as the daily median for each country considered, normalized and computed as shown in Section 2 of the paper.

Figure 4 clearly shows the decreasing pattern from the first period to the second, with an average decrease of 8.2% in the air pollution index, while from the second period to the third the decrease was around 3.2%.

COVID-19 restrictions had the biggest positive impact regarding air pollution in Ukraine, Portugal, Bosnia and Herzegovina, Hungary, Italy and Austria, although Bosnia and Herzegovina, Hungary and Italy remained the most air polluted countries in Europe in late 2020. In contrast, Bulgaria, Romania and Iceland are the top cleanest countries regarding tropospheric air emissions. Before and after maps, presented in Figure 4, expose, in addition to the most air polluted countries within Europe, the most positively affected countries, with the most visible impact on Ukraine. If we relate the changes to restrictions put in place over the specified period of time, Ukraine, Bosnia and Herzegovina and Austria were in the top positively affected countries considering the restrictions put in place.

The results show that nitrogen dioxide had the biggest decrease from the first period to the last (from March to October), representing approx. 20.69% of the initial value, while particle matter 2.5 and carbon monoxide followed with an average decrease of 13.64% and 11.79%, respectively, from the first period to the last.

**Figure 5:**
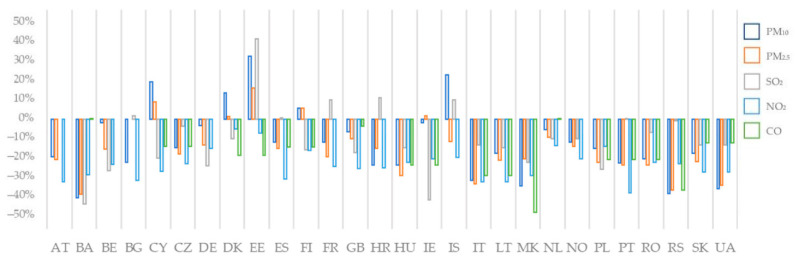
Changes in pollutants concentration in urban air at the surface level.

Changes in breathable air parameters are presented below, in Figure 5, as relative values from 1 March–30 May to 1 September–30 October, for each country and species analyzed:

(9) $$\mathrm{Changes}\;\mathrm{in}\;\mathrm{pollutant}\;P=\frac{{\mathrm{avg}\;}_{1}\left[\;Z\;\right]}{{\mathrm{avg}\;}_{0}\left[\;Z\;\right]}\times 100-100\%$$

where avg_0_ [P] represents the average of the daily median of the P pollutant for the period 1 March–30 May and avg_1_ [P] represents the average of the daily median of the P pollutant for the period 1 September–30 October.

All species that were studied in the present paper suffered individual drops, or a slightly stable concentration from March to October.

From Figure 5 we can observe that the PM_10_ concentration dropped in the analyzed period for all the countries, except for Cyprus, Estonia, Iceland and Denmark, where the concentrations of PM_10_ in urban air had an increasing pattern from March to October 2020. Moreover, Estonia and Iceland had increasing patterns in both SO_2_ and PM_2.5_, while Cyprus’ concentration of SO_2_ decreased over the analyzed period. North Macedonia, Bosnia and Herzegovina, Serbia, Italy and Ukraine registered significant drops in all the analyzed pollutants, except for Bosnia and Herzegovina’s changes in CO concentrations. On the other side, Denmark and Finland had increasing patterns in PM_10_ and PM_2.5_ concentrations, but drops in SO_2_, NO_2_ and CO. While NO_2_ had decreasing trends for all of the countries, CO and SO_2_ registered drops or maintained the same concentrations over the analyzed period, except for in Estonia, France and Croatia, where significant or small increases for SO_2_ were registered. PM_10_ and PM_2.5_ registered decreasing trends for the intercontinental countries, while they showed growth for the coastal, peninsular or insular ones.

The correlation of the air pollution index with the changes in industrial production and manufacture is highly important in order to understand the impact of each industry and manufacture economic activity as a source for air pollution. Thus, Table 3 presents correlations among the changes in the main industries and manufacture activities from 2019 to 2020 and the changes in air pollution during the same period.

It can be seen from Table 3 that the manufacture of textiles, motor vehicles, trailers and semi-trailers, and rubber and plastic products along with activities of households as employers and undifferentiated goods- and services-producing activities of households for their own use are the activities most correlated with air pollution among industrial and manufacturing activities.

### 3.2. Meteorological Analysis

**Figure 6:**
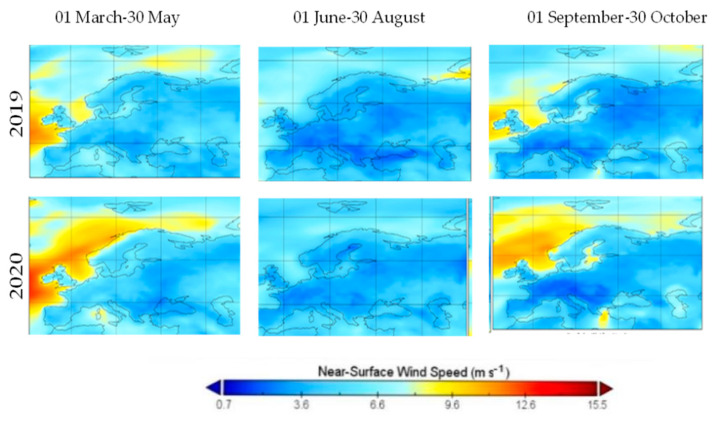
Mean of ground level wind speed over Europe in three periods, 2019 and 2020.

Wind speed in spring 2020 exceeded the values from the same period in 2019, while the values from summer suffered a regression from 2019 to 2020, especially in the Iberian Peninsula. In September wind speed all over Europe presented a higher value in 2019 than 2020, with an exception in the Iberian Peninsula, while in the rest of autumn the upper trend from 2019 remained present, as shown in Figure 6. Wind speed was found to be negatively associated with the concentration of air pollutants, as shown in [16], with a higher air pollution being expected in 2020 than in 2019. The results are opposite to this statement, and thus it is reasonable to argue that the lowered value of air pollutants in 2020 had its source in other causes than the state of the wind.

**Figure 7:**
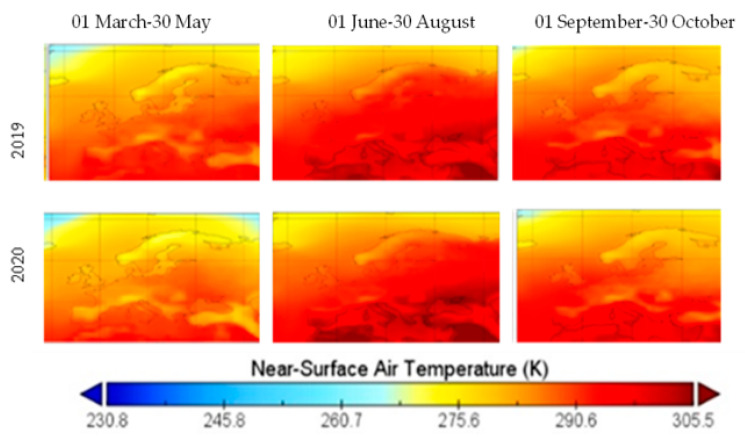
Mean of near-surface air temperature over Europe in three periods, 2019 and 2020.

Mean temperature over Europe registered lower values in 2020 than 2019 in spring, summer and autumn, with an exception in July, when the Balkan region registered higher values in 2020 than 2019, as shown in Figure 7. While cold weather tends to foster the accumulation of PM and CO and make pollutants more visible, warm weather increases the amount of O_3_ concentration, along with PM_2.5_, PM_10_ and CO, when associated with natural forest fires. Moreover, CO tends to decrease when temperatures are high. The lower value of near-surface temperatures justifies, in part, the higher visibility of PM_2.5_, PM_10_ and CO.

**Figure 8:**
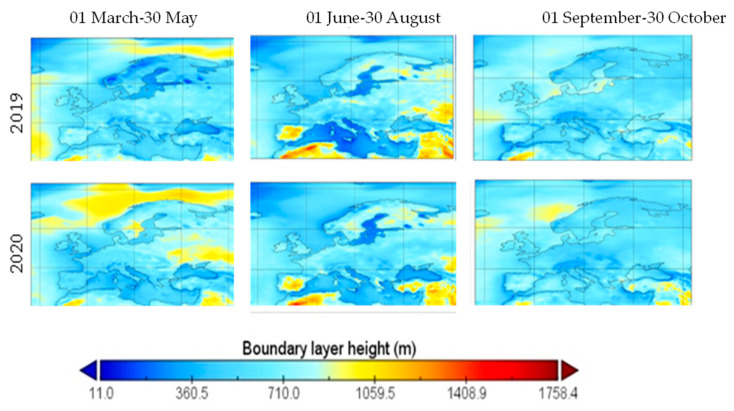
Average of the boundary layer height over Europe in three periods, 2019 and 2020.

The average of the boundary layer height was lower in the months of April-August in 2019 than in April-August in 2020, while presenting comparable values in autumn, with an exception for Scandinavia and the Baltics, where the boundary layer height was lower in 2019 than 2020, as shown in Figure 8. Boundary layer height and the ventilation coefficient are negatively correlated with the concentration of air pollutants, as shown in [17] for PM_2.5_ and black carbon.

**Figure 9:**
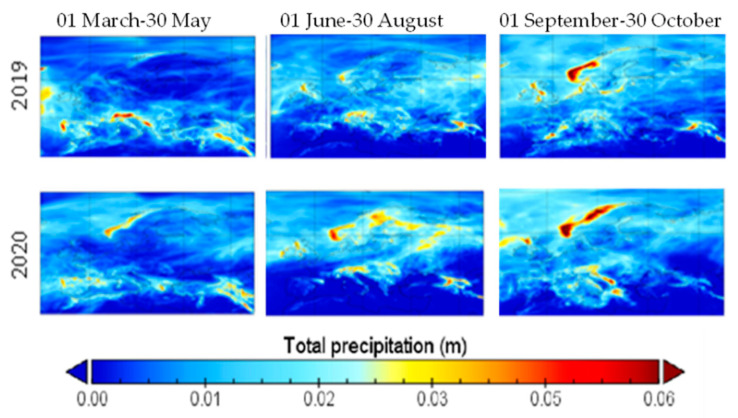
Mean of total precipitation over Europe in three periods, 2019 and 2020.

Total precipitations of 2020 exceeded the values from 2019 in June and July, and were exceeded by the ones from 2019 in April and May, as shown in Figure 9. Total precipitations are negatively associated with the concentration of NO_2_, CO and PM when controlling for wind speed, as shown in [16], while they are positively correlated with SO_2_.

### 3.3. Individual Air Pollutants Analysis

**Figure 10:**
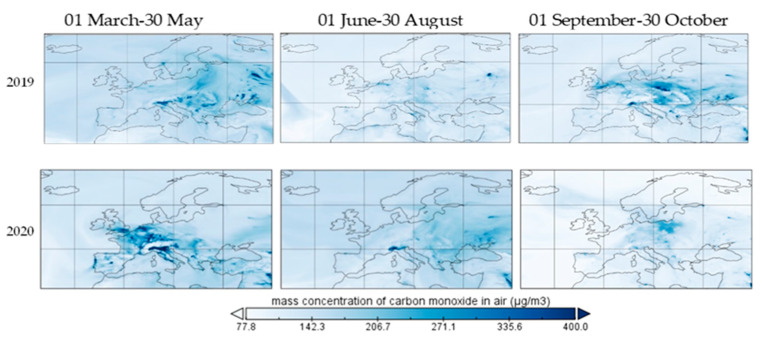
Carbon monoxide over Europe during restriction period, 2019 and 2020.

In Figure 10, the mass concentration of carbon monoxide over Europe on three different dates is provided, covering the period before and after COVID-19 lockdown. The concentrations presented are averages of hourly data, as provided by the Copernicus Atmosphere Monitoring Station (CAMS).

Although the wind speed was more intense in April and May 2020 over Europe than in the same period of 2019, in July–September 2020 the intensity was set under the value of the same period of 2019, and the concentration of CO maintained a downward slope, in comparison with the same period of 2019, when the concentration was rising in the autumn of 2019. In [16], wind speed is negatively associated with the concentration of CO, when controlling for temperature, precipitation and relative humidity, with values ranging between −0.164 and −0.120, while temperature is negatively correlated when controlling for wind speed and air pressure (−0.225 and −0.272) and positively correlated when controlling for precipitation and relative humidity (0.220 and 0.197). Though mean temperatures in the late spring, summer and first period of autumn of 2019 were higher than in the same period of 2020, the concentration of CO in 2020 was not exceeded on the basis of colder weather, as expected. The boundary layer height was lower in April–July 2019 than in the same period of 2020; thus, it is expected for the concentration of CO to decrease from 2019 to 2020. Total precipitations of May and April 2019 surpass the amount from the same period of 2020, but in June–July the situation was reversed; thus, it is expected, by this condition, for the CO concentration to be lower in May and April of 2020 compared with the same months of 2019, and the opposite is expected in the June–July period.

Our analysis shows that carbon monoxide was cleaned up at the ground-level layer in Europe from March to October, with an approximately 12% decrease based on a reduction of activities that engage fossil fuel combustion, which is the main source of CO, up to 95%, as reported in [18]. Although fossil fuel combustion may primarily consist of outdoor activities, such as traffic, industry or power plants, some indoor activities may continue emitting the gas. For example, heaters engaged in cooking or heating and smoking can produce CO [19], although indoor pollution does not reach the industrial levels [13]. In addition to its positive effects, such as enhancing plant growth in labs, mediating efficacy in pulmonary hypertension and acute liver failure when associated with NO, health benefits in animals in different pathologies, potential anti-inflammatory effect, anti-apoptotic effect, positive effects on blocking CO proliferation and anti-aggregatory properties, carbon monoxide can cause serious poisoning due to its complex bond with hemoglobin, thus reducing the capacity of O_2_ absorption [19]. Short-term health effects of CO may include headache, dizziness, weakness, nausea, vomiting and even loss of consciousness and death from poisoning [19]. Moreover, visual perception, audition, sensorimotor performance and vigilance are weakened after a short exposure to CO. Long exposure to CO can lead to the development of long-term neurological symptoms, changes in memory, sleep changes, vision, smell and direction changes, anxiety, psychomotor disfunction and balance problems. Moreover, subtle effects on the brain after prolonged low exposure were obtained in [20]. It may cause more than 50% of fatal poisonings [21]. From the first period to the third one, carbon monoxide showed a 12% drop in outdoor concentrations on the ground-level as measured by outdoor stationary monitoring stations along most important cities in Europe. Figure 10 presents the most cleaned areas of tropospheric CO above Europe, showing a pattern of industrial carbon monoxide, with a low impact of household sources on outdoor air pollution. Figure 10 shows that ground-level carbon monoxide does not exceed the 9 ppm per 8 h exposure (approx. 10,310 μg/m^3^) in the US EPA standard or 10 mg/m^3^ (10,000 μg/m^3^) in EU standards.

**Figure 11:**
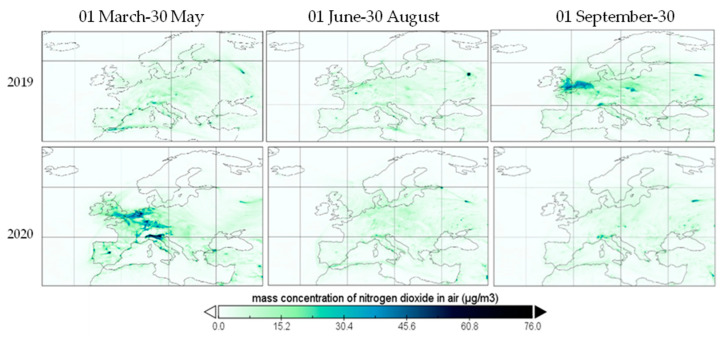
Nitrogen dioxide over Europe during restriction period, 2019 and 2020.

In Figure 11, the mass concentration of nitrogen dioxide over Europe on three different dates is provided, covering the period before and after COVID-19 lockdown from spring 2020. The concentrations presented are averages of hourly data, as provided by the Copernicus Atmosphere Monitoring Station (CAMS).

The mass concentration of nitrogen dioxide registered a major regression of 20.69% from March to June 2020 compared to the same period of the previous year, over Europe, as a result of a higher value of wind speed in April and May 2020 and boundary layer height in late spring and summer 2020, compared to 2019, in addition to the transportation and industrial activities reduction starting from March 2020. Moreover, in [22], a study conducted in the Lombardia Region in Italy, based on a multivariate regression, it was concluded that wind speed negatively affects the NO_2_ concentrations with an approximatively 6% reduction. In a study conducted in Bangladesh, [23], wind speed was negatively corelated with nitrogen dioxide with a correlation of −0.37, while temperature and humidity presented nonsignificant correlations. Although total precipitation was lower in late spring of 2020, compared to 2019, the trend was reversed in October, thus improving the air quality in comparison with the same period of 2019 by negatively correlating with NO_2_ concentration [16]. In [22], it was found that both temperature and precipitation have no significant effect on NO_2_ pollutant concentrations. Moreover, in [24], boundary layer height had the most significant influence over NO_2_ concentration (18 ± 6%) followed by the surface wind speed (12 ± 5%).

Results show that nitrogen dioxide, the most important traffic-related pollutant, suffered a visible drop from the first period to the third one, based on a significant reduction of human mobility, expressed also by an oil demand reduction of 5%, by restricting aviation and terrestrial mobility (accounting for 60% of global oil demand) [13]. Thus, NO_2_ recorded an approximately 21% reduction in terrestrial concentration, with a prolonged positive effect of the lockdown period and mobility restrictions. Health effects caused from exposure to NO_2_ include respiratory system irritation and disease, coughing, wheezing, dyspnea, bronchospasm and eyes, throat and nose symptoms with limited exposure. NO_2_ exposure can lead to pulmonary edema when a person is exposed to large concentrations (>0.2 ppm) or chronic lung disease when long-term exposure is involved. Other than during fossil fuel combustion from transportation and aviation, nitrogen is also released during synthetic fertilization, deforestation and biomass burning and from natural soils or oceans [25], of which only oceans possess a significant impact over coastal urban areas [26]. Although the biggest percentage of nitrogen dioxide emissions comes from the Haber–Bosch fertilization process [25,26], urban emissions are dominated by traffic-related activities [27]. As restrictions forced a human mobility reduction of up to 23 points in March 2020, in Europe, all traffic-related pollutants dropped along urban areas across Europe, with the most significant impact on nitrogen dioxide. While annual nitrogen dioxide levels were elevated in developed countries such as Italy, Germany and France before lockdown, surpassing the annual limit of Europe, after lockdown the general level of NO_2_ stabilized around 15 μg/m^3^, below the US EPA annual limit of 99.73 μg/m^3^ and EU annual limit of 40 µg/m^3^, as shown in Figure 11.

**Figure 12:**
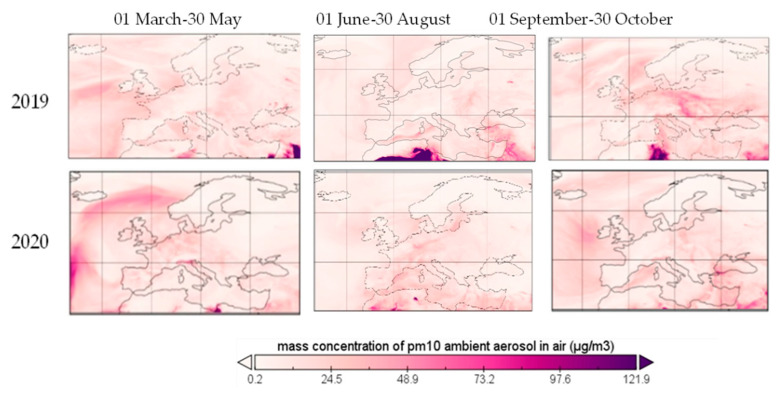
PM_10_ over Europe during restriction period, 2019 and 2020.

**Figure 13:**
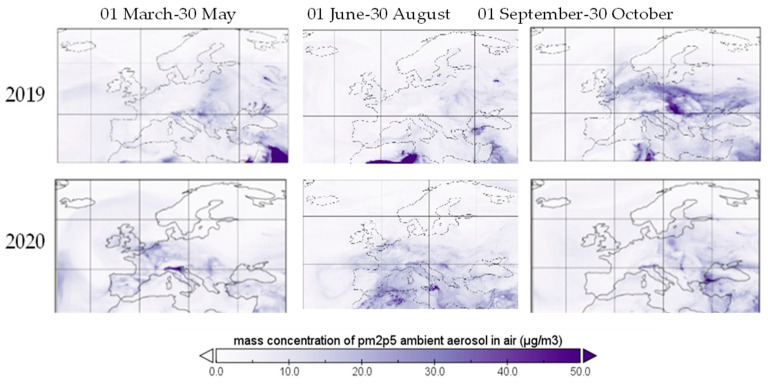
PM_2.5_ over Europe during restriction period, 2019 and 2020.

In Figure 12 and Figure 13 the mass concentrations of PM_10_ and PM_2.5_ over Europe in three different periods are provided, covering the period before and after COVID-19 lockdown. The concentrations presented are averages of hourly data as provided by the Copernicus Atmosphere Monitoring Station (CAMS).

As a result of the negative association with temperature, the values of PM_10_ and PM_2.5_ from 2020 are below the ones from 2019, while the mean temperature from 2019 exceeded the near-surface air temperature at the tropospheric level from 2020. In October both PM_10_ and PM_2.5_ registered higher values in 2019 than the rest of the year, as a result of a lower mean temperature, but the spike was not present in 2020, when the decreasing trend was maintained. In contrast, in [22] it was found that both temperature and precipitation have no significant effect on PM_10_ pollutant concentrations. The values were higher in winter and lower in summer, in both periods. Furthermore, in [22], it was concluded that wind speed negatively affects the PM_10_ concentrations with values ranging from −13.89 to −6.97. Moreover, in [16], wind speed is negatively correlated with PM_2.5_ when controlling for temperature, relative humidity, air pressure and precipitation with values ranging between −0.167 and −0.055. In a study conducted in Bangladesh, [23], wind speed is negatively corelated with PM_2.5_ with a value of −0.58. In the same study, temperature is negatively associated with PM_10_ and PM_2.5_ when controlling for wind speed and air pressure, with values ranging between −0.406 and −0.251, while being positively associated when controlling for precipitation (0.255 and 0.238). Total precipitation is negatively associated with both PM_10_ and PM_2.5_ when controlling for wind speed, temperature, relative humidity and air pressure with values between −0.605 and −0.323.

Particle matter is a pollutant caused by fossil fuel combustion, industrial and agricultural activities, in direct form, or within the process of transformation of NO_x_, SO_2_ or other chemical compounds [28], being directly linked to the traffic level in urban areas [29]. Natural sources, such as volcanoes, dust storms, forest fires, living vegetation or sea spray count for a small percentage in urban areas, with the exception of sea spray, which is the largest source for coastal urban PM_10_ [30]. Health effects are related to dyspnea (on short-term exposure) and cardiovascular disease and infant mortality on long-term exposure to PM_2.5_. Heath effects of long exposure to PM_10_ and PM_2.5_ include respiratory diseases, afflictions of the immune system, cancer, diabetes or other toxic effects and short exposure impact may consist of chest discomfort, chest pain, coughing and wheezing [19]. Along with these negative effects, PM can work as an adjuvant for allergic sensitization [31]. Restrictions on human mobility, over the first period, generated an almost 14% PM_2.5_ reduction, based on the reduction of the combustion of coal, oil and gasoline by almost 3.8% in the first trimester [13] and the reduction of the transformation of other chemical pollutants, due to the reduction of already mentioned gasses together with the reduction in the erosion of pavements and abrasion of multivehicle components as a result of transportation restrictions. PM_10_ registered a 10% decrease in urban areas, mostly based on suspension of soil reduction when agricultural and industrial activities or construction and transportation dropped, while ocean spray remained at the same markers, within its annual cycle, in the analyzed period. Thus, Figure 12 and Figure 13 show the decreasing trend of both PM_10_ and PM_2.5_ in terrestrial areas within the European Union. PM_10_ did not exceed the NAAQS limit for 24 h in all the analyzed period, all the concentrations being under 150 μg/m^3^, with Bosnia and Herzegovina and North Macedonia having the highest concentrations in the first days of March. Furthermore, PM_10_ did not exceed the EU annual standards of 40 µg/m^3^ in the whole period, except for Bosnia and Herzegovina, North Macedonia and Serbia. All the PM_2.5_ concentrations exceeded the 24 h EPA standard of 12 μg/m^3^ and the annual standard of 35 μg/m^3^ in the analyzed period, except for Bulgaria. Moreover, PM_2.5_ exceeded the EU standards of 25 μg/m^3^ for most of the analyzed countries in the analyzed period.

**Figure 14:**
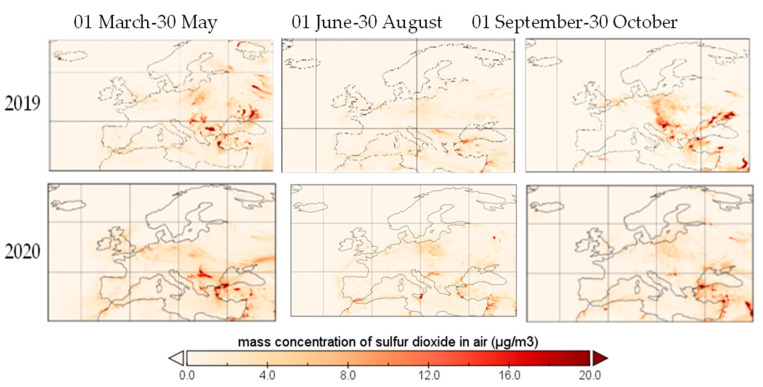
Sulfur dioxide over Europe during restriction period, 2020.

In Figure 14, the mass concentration of sulfur dioxide over Europe on three different dates is provided, covering the period before and after COVID-19 lockdown from spring 2020. The concentrations presented are averages of hourly data as provided by the Copernicus Atmosphere Monitoring Station (CAMS).

Sulphur dioxide maintained the decreasing trend from spring to autumn 2020, in opposition with 2019, when the concentration presented a spike in autumn. Total precipitations were higher in October 2020 than in October 2019, so the concentration of SO_2_ should be lower in 2020 than in 2019 (in the same period, as they are negatively associated with values ranging between −0.378 and −0.215 [16]). Among the health effects of SO_2_, irritation to the respiratory system, penetration deep into the lungs, the introduction of respiratory disease, bronchospasm/pulmonary edema, eye symptoms and cardiovascular disease were found in [32], while skin redness was mentioned in [33].

Sulfur dioxide suffered a reduction of 8.38% in ground-level emissions. The main source of sulfur dioxide, such as power plants and other fossil fuel sources, along with oil refineries and natural gas refineries, reported a reduction in activity based on general energy demand reduction, and sulfur dioxide followed the same trend, while other sources still settled at the same scale, such as biomass burning [34] or smelters, which are some of the largest sources of SO_2_ [35]. Moreover, individual facilities are not stringently considered as hotspots of sulfur dioxide emissions, as a recent study on the Middle East exposed [35]. Sulfur dioxide did not exceed the EU limit of 125 μg/m^3^ in the analyzed period, as shown in Figure 14, nor the US EPA annual standard of 0.3 ppm.

### 3.4. ARX Model

Table 4 presents the correlation coefficients between the restriction index constructed as an aggregated index of austerity and the analyzed pollutants. Thus, between the restriction index and air quality we observe a very strong relationship with a correlation coefficient of 0.76 (with a significance value of 0.000). All correlations present a strong and direct relationship among the variables.

In validating the alternative hypothesis that confirms the significant influence of human mobility restrictions over urban air quality, we used a paired *t*-test that concluded with a *p*-value of 0.00 under the considered significance level of 0.05. We assumed the normal distribution of sample means by the central limit theorem, taking into consideration that we used over 300,000 data points.

To understand the behavior of air pollution, we constructed an autoregression with exogenous variable (ARX). The best fitted model is constructed upon the last four periods (x_t−1_, x_t−2_, x_t−3,_ x_t−4_) and the last pollution index (y_t−1_), as shown by Equation (10). The function took the following form:

(10) $$y_{t}={\alpha y}_{t-1}+\beta_{1}x_{t}+\beta_{2}x_{t-1}+\beta_{3}x_{t-2}+\beta_{4}x_{t-3}+\beta_{5}x_{t-4}+\varepsilon_{t}$$

where α and β are coefficients; y is the dependent variable, air pollution; x is the independent variable, the restriction index; and ε are the residuals.

**Figure 15:**
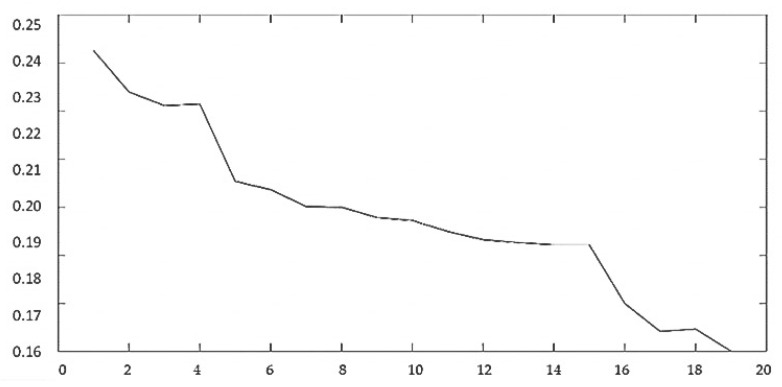
Forecasting air pollution by maintaining maximum restrictions for a four-month period.

We used the autoregressive function to forecast the air pollution index in the next four months, considering the restrictions to be at their maximum value. We took into consideration a value of restriction of 22.8, being the maximum value of the restriction index in the analyzed period. The forecast presumes the same behavior of air pollution based on the last historical data on air pollution and restriction. Figure 15 presents the behavior of air pollution, under an ARX model, taking into consideration the last period for the pollution index and the last four periods of the restriction index. The left scale of the graphic shows the air pollution index value for each of the periods analyzed, with a minimum of 0.16 and a maximum of 0.25. The horizontal axis shows the period considered (from 0 to 20). It is clearly shown in Figure 15 that urban air pollution may be reduced below historical values, if human mobility restriction is maintained at the maximum levels.

## 4. Discussion

The present study aimed to argue the impact of COVID-19 restrictions over air pollution dynamics, such as the air pollution index in urban areas and changes in tropospheric air pollutants or pollution hotspots during or after the lockdown period. Results show a direct, significant relationship between restrictions and air pollution indicators.

Overall, air pollution dropped by 12%, on average, with a local maximum in Ukraine (27% drop in air pollution), Croatia (with a 29% drop from March to October), Bulgaria, Lithuania and Portugal.

The results are supported by other findings, as in [36], where CO, NO_2_, PM_2.5_ and PM_10_ decrease and air quality improvement were associated with the lockdown period. Moreover, decreased NO_2_ concentrations were reported in Italy, France and Poland [37,38,39]. Also, due to several measures taken by the government, there were reported decreases in CO, PM_2.5_ and PM_10_ in the US, Italy and Poland [40]. NO_2_ reduction was also reported in Italy, China, India and the US [41] due to oil demand reduction. The same sources mention clean waters where water traffic was abundant before lockdown. A drop of 47–55% in NO_2_ and CO emissions was reported in European countries in [42]. Reduction in NO_2_ emissions in Italy, China, the US, Spain and France were obtained by satellite images in [43]. Moreover, Ref. [44] reported a reduction of 40% in NO_2_, with oil and coal as the most significant NO_2_ sources. Furthermore, as traffic was reduced by almost 70% in the UK during lockdown, there was a reported 38.3% drop in NO_2_ and 16.5% drop in PM_2.5_ [45]. Similar results on CO and NO_2_ were found in Italy [46]. As discussed in the present paper, general energy demand reduction contributes to air quality improvement. An interesting distinction was found between PM_2.5_ and PM_10_ changes, as ocean spray still remains an important source of PM_10_, with some studies supporting the same findings [47]. NO_2_, SO_2_ and PM reduction in India was also reported in [48]. Moreover, an improvement in air quality index was found in northern China [49], with a decrease in SO_2_, PM_2.5_, PM_10_, NO_2_ and CO (a drop of 6.76%, 5.93%, 13.66%, 24.67% and 4.58%, respectively). These results are consistent with our findings. A significant reduction of PM_2.5_ was also found in India, as shown in [50], while particle matter, NO_2_ and CO registered a drop during lockdown in the megacity Delhi, the actual most polluted city worldwide, with an approximate 40–50% increase in air quality [51], and in [44] particle matter dropped by 10%.

Other studies reported serious causalities among air pollutants and virus transmission, as in [36] it is stated that nitrogen dioxide is a promotor of COVID-19 transmission. For example, a significant increase in incidence rates is related to 1 μg/m^3^ growth in NO_2_, and a smaller increase is related to 1 μg/m^3^ growth in PM_2.5_ [52]. In contrast, in [53], a weak association between O_3_ and PM_2.5_ with COVID-19 incidence rate was reported. Similar results were found in [54], where PM_10_ and NO_2_ were also significantly related to the risk of COVID-19 diagnosis.

In 2017, air pollution caused almost 1,200,000 deaths in India, and the same in China [44], with a total of 7 million deaths worldwide [55] in association with respiratory or pulmonary diseases; thus, ground-level air clean-up comes as a necessity in the global context. Moreover, COVID-19 had a 1.4% mortality rate [56], while air pollution registered a 7.6% mortality rate in in 2016 [57]. Furthermore, in 2019 a total of 441,998 premature deaths were associated with tropospheric air pollution across Europe, of which 307,000 were associated with PM_2.5_, 40,400 with NO_2_ and 16,800 with O_3_ [58]. According to the European Centre for Disease Prevention and Control, in Europe a total of 1,111,693 deaths were attributed to COVID-19 disease in 2020. Our data show that approximatively 42,000 premature deaths were avoided due to a reduction in PM_2.5_, while 8000 premature deaths were avoided due to a reduction in NO_2_, far beyond the number of deaths reported in relation with COVID-19 disease. In addition, an increased trend in waste fires, including biomass, was mentioned in [44] based on people’s incapacity to properly handle the waste.

The present study was conducted on a continental region, including 28 countries, on a time perspective of 10 months, with daily data on each air pollutant and restriction being handled, with a total of over 300,000 data points that were imputed for missing values, normalized and aggregated to construct air quality and restriction indexes. Limitations of the study include the lack of data on methane and CO_2_, which is necessary to pursue for a more exhaustive approach, and the lack of a quantitative meteorological analysis and the implied uncertainties related to it.

Future directions may concentrate on widening the geographical area upon which the present study was conducted by including the global area and pursuing a global forecasting model of tropospheric air pollution on behalf of the intensity of human activity and mobility.

## 5. Conclusions

Before pandemic restrictions were put in place, ground-level air pollution was a serious problem regarding human health, consisting of a 7.6% mortality rate in 2016 [57], while in the lockdown period air pollution dropped by 12%. All pollutants registered declines, such as SO_2_, which registered an 8.58% decrease; CO, which registered an 11.94% decrease; PM_2.5_, which dropped by 13.64%; and PM_10_, which dropped by 10.63%. NO_2_ presented the most significant drop of around 20.69%. All measurements were taken from urban monitoring stations in major cities across Europe.

The main causes of air quality improvement consist of energy demand reduction and transportation, industrial and agricultural activities reduction, although ocean spray remains an important source of PM_10_, explaining the small reduction of PM_10_ in comparison with PM_2.5_. Other studies showed similar results in the same or different regions.

To observe the future behavior of air pollution, we constructed an autoregression with exogenous variable and found that the influence of restrictions is prolonged for four periods on best fit regression, with a forecast of another 3% drop in air pollution if restrictions maintain a maximum value.

The best improvement was found in Ukraine, Austria and Bosnia and Herzegovina, while almost all analyzed pollutants had concentrations below US EPA and EU standards.

## Figures and Tables

**Table 1:** Data sources.

Name	Parameter	Source	Unit	Vertical Level	Frequency of Available Data	Usage
Sulphur dioxide	SO_2_	Environmental Protection Agency repository (EPA)	Parts per million (PPM)	Surface (0 m above ground)	Daily	Air pollution index (API) and air quality index (AQI)
Carbon monoxide	CO	EPA	Parts per billion (PPB)	Surface	Daily	API; AQI
Nitrogen dioxide	NO_2_	EPA	PPB	Surface	Daily	API; AQI
Particle matter < 2.5 microns	PM_2.5_	EPA	μg/m^3^ (micrograms per cubic meter air)	Surface	Daily	API; AQI
Particle matter < 10 microns	PM_10_	EPA	μg/m^3^	Surface	Daily	API; AQI
Sulphur dioxide concentration in NetCDF	SO_2_	NASA Orbiting Carbon Observatory 2 product, through Copernicus Atmosphere Monitoring Service (OCO2, CAMS)	μg/m^3^	Surface	Hourly	Map representation
Carbon monoxide concentration in NetCDF	CO	OCO2, CAMS	μg/m^3^	Surface	Hourly	Map representation
Nitrogen dioxide concentration in NetCDF	NO_2_	OCO2, CAMS	μg/m^3^	Surface	Hourly	Map representation
Particle matter < 2.5 microns concentration in NetCDF	PM_2.5_	OCO2, CAMS	μg/m^3^	Surface	Hourly	Map representation
Particle matter < 10 microns concentration in NetCDF	PM_10_	OCO2, CAMS	μg/m^3^	Surface	Hourly	Map representation
Ground level wind speed	WIND	ERA5 satellite through CAMS	Meters/second (m/s)	Surface	Hourly	Map representation and meteorological analysis
Near-surface air temperature	TEMP	ERA5 satellite through CAMS	Kelvin degrees (K)	Surface	Hourly	Map representation and meteorological analysis
Boundary layer height	-	ERA5 satellite through CAMS	M	Surface	Hourly	Map representation and meteorological analysis
Total precipitation	PRECIP	ERA5 satellite through CAMS	M	Surface	Hourly	Map representation and meteorological analysis
Restrictions in COVID-19 pandemic	RES	European Centre for Disease Prevention and Control repository	-	-	Daily	Restriction index (RES)
Air emission accounts	-	NACE rev. 2 classification from Eurostat repository	-	-	Annually	Identifying the weights for the restriction index
National accounts aggregates by industry	-	Eurostat	-	-	Annually	Correlation of the air pollution index with the changes in industrial production and manufacture

**Table 2:** Periods used in the analysis.

Period	Usage	Reason
14-day period (approx. bimonthly)	Construction of API, AQI and RES index, correlations and ARX approach	The period of incubation for COVID-19 symptoms could extend to 14 days; governments usually took into consideration a two-week lockdown period.
1 March–30 May, 1 June–30 August and 1 September–30 October	Maps of SO_2_, CO, NO_2_, PM_2.5_ and PM_10_ over Europe; Air pollution and restrictions over Europe; Changes in pollutants concentration in air; Maps of ground level wind speed, near-surface air temperature, boundary layer height and total precipitation.	Analysis of the seasonal changes of the air pollution components over Europe.

**Table 3:** Pearson correlation between changes in production in the main industries and manufacture activities and changes in urban air pollution.

	*p*-Value	Pearson Correlation	Industry and Manufacture Economic Activities
1	<10%	0.29542	Manufacture of textiles, wearing apparel, leather and related products
2	<10%	0.29942	Manufacture of rubber and plastic products
3	<5%	0.47511	Manufacture of motor vehicles, trailers and semi-trailers
4	<5%	0.34065	Activities of households as employers; undifferentiated goods- and services-producing activities of households for own use

**Table 4:** Correlation of restriction index with air pollutants (US EPA standard).

**Correlation** **of Restriction Index with**	**Air Quality**	**PM_10_**	**PM_2.5_**	**SO_2_**	**NO_2_**	**CO**
	76.65% *	74.26% *	71.53% *	68.01% *	64.06% *	64.96% *

* Sig. under 0.05.

## Data Availability

Data are available in a publicly accessible repository that does not issue DOIs. Publicly available datasets were analyzed in this study. These data can be found here: Environmental Protection Agency repository: https://aqicn.org/data-platform/covid19/ (accessed on 5 May 2021), Copernicus Atmosphere Monitoring: https://ads.atmosphere.copernicus.eu/cdsapp#!/dataset/cams-europe-air-quality-forecasts?tab=overview (accessed on 5 May 2021), ERA5 meteorological data: https://cds.climate.copernicus.eu/cdsapp#!/dataset/reanalysis-era5-single-levels?tab=overview (accessed on 6 August 2021) European Centre for Disease Prevention and Control—restriction data: https://www.ecdc.europa.eu/en/publications-data/download-data-response-measures-covid-19 (accessed on 5 May 2021), Air emissions accounts by NACE rev. 2 activity: http://appsso.eurostat.ec.europa.eu/nui/show.do?dataset=env_ac_ainah_r2&lang=en (accessed on 15 May 2021)**,** NACE rev. 2 activity methodology and definitions of activities: https://ec.europa.eu/eurostat/web/products-manuals-and-guidelines/-/ks-ra-07-015 (accessed on 15 May 2021), US EPA air quality methodology: https://www.airnow.gov/sites/default/files/2020-05/aqi-technical-assistance-document-sept2018.pdf (accessed on 5 May 2021), and Data on industry and manufacturing activities and their contribution to national value added: https://ec.europa.eu/eurostat/databrowser/view/nama_10_a64/default/table?lang=en (accessed on 21 July 2022).

## Glossary of Symbols

**Symbol**	**Description**
NO_2_	Nitrogen dioxide
SO_2_	Sulphur dioxide
CO	Carbon monoxide
PM_2.5_	Particle matter under 2.5 microns diameter
PM_10_	Particle matter under 10 microns diameter
NASA	The National Aeronautics and Space Administration
COVID-19	Coronavirus Disease 2019
SARS-CoV-2	Severe acute respiratory syndrome coronavirus 2
WHO	World Health Organization
N95 masks	Respirators that filter out 95 percent of airborne particles
EPA	United States Environmental Protection Agency
PPB	Parts per billion
OCO2	NASA Orbiting Carbon Observatory 2 product
CAMS	Copernicus Atmosphere Monitoring Service
ERA5 satellite	The fifth generation ECMWF atmospheric reanalysis of the global climate
NACE rev. 2	Statistical classification of economic activities in the European Community
US EPA standard	United States Environmental Protection Agency data standards
NAAQS	National ambient air quality standard
I_P_	Air pollution score for each pollutant
C_P_	The period average truncated concentration for each pollutant
BP_Hi_	The concentration breakpoint first to exceed the C_P_
BP_Lo_	The concentration breakpoint first below the C_P_
I_Hi_	The AQI values corresponding to BP_Hi_
I_Lo_	The AQI values corresponding to BP_Lo_
ARX	Autoregressive model with exogenous input
API_change_	Air pollution index changes
API_period y_ (X)	The air pollution index for each of the three periods: 1 March–30 May, 1 June–30 August and 1 September–30 October
X	Each country taken into consideration
P	Pollutant
US	United States
PPM	Parts per million
NetCDF	Network Common Data Form

## References

[1] Glencross D.A., Ho T.R., Camiña N., Hawrylowicz C.M., Pfeffer P.E. (2020). Air Pollution and Its Effects on the Immune System. Free Radic. Biol. Med..

[2] Williams D.D.R. Earth Fact Sheet. https://nssdc.gsfc.nasa.gov/planetary/factsheet/earthfact.html.

[3] Vallero D. (2008). Fundamentals of Air Pollution.

[4] Moraru R.I., Băbuţ G.B., Cioca L.I. Study of Methane Flow in Caved Goafs Ajacent to Longwall Faces in Valea Jiului Coal Basin. Proceedings of the 13th International Multidisciplinary Scientific GeoConference SGEM.

[5] Yuki K., Fujiogi M., Koutsogiannaki S. (2020). COVID-19 Pathophysiology: A Review. Clin. Immunol..

[6] Wang P., Chen K., Zhu S., Wang P., Zhang H. (2020). Severe Air Pollution Events Not Avoided by Reduced Anthropogenic Activities during COVID-19 Outbreak. Resour. Conserv. Recycl..

[7] Baloch S., Baloch M.A., Zheng T., Pei X. (2020). The Coronavirus Disease 2019 (COVID-19) Pandemic. Tohoku J. Exp. Med..

[8] Hammett E. (2020). How Long Does Coronavirus Survive on Different Surfaces?. BDJ Team.

[9] Chu D.K., Akl E.A., Duda S., Solo K., Yaacoub S., Schünemann H.J., Chu D.K., Akl E.A., El-harakeh A., Bognanni A. (2020). Physical Distancing, Face Masks, and Eye Protection to Prevent Person-to-Person Transmission of SARS-CoV-2 and COVID-19: A Systematic Review and Meta-Analysis. Lancet.

[10] Kraemer M.U.G., Yang C.-H., Gutierrez B., Wu C.-H., Klein B., Pigott D.M., du Plessis L., Faria N.R., Li R., Hanage W.P. (2020). The Effect of Human Mobility and Control Measures on the COVID-19 Epidemic in China. Science.

[11] Saglietto A., D’Ascenzo F., Zoccai G.B., De Ferrari G.M. (2020). COVID-19 in Europe: The Italian Lesson. Lancet.

[12] Rothan H.A., Byrareddy S.N. (2020). The Epidemiology and Pathogenesis of Coronavirus Disease (COVID-19) Outbreak. J. Autoimmun..

[13] IEA (2020). Global Energy Review 2020.

[14] Eurostat (2008). NACE Rev. 2—Statistical Classification of Economic Activities in the European Community.

[15] Cioca L.I., Ivascu L., Rada E.C., Torretta V., Ionescu G. (2015). Sustainable Development and Technological Impact on CO_2_ Reducing Conditions in Romania. Sustainability.

[16] Liu Y., Zhou Y., Lu J. (2020). Exploring the Relationship between Air Pollution and Meteorological Conditions in China under Environmental Governance. Sci. Rep..

[17] Sujatha P., Mahalakshmi D.V., Ramiz A., Rao P.V.N., Naidu C.V. (2016). Ventilation Coefficient and Boundary Layer Height Impact on Urban Air Quality. Cogent Environ. Sci..

[18] Transportation Research Board and National Research Council (2003). Managing Carbon Monoxide Pollution in Meteorological and Topographical Problem Areas.

[19] Manisalidis I., Stavropoulou E., Stavropoulos A., Bezirtzoglou E. (2020). Environmental and Health Impacts of Air Pollution: A Review. Front. Public Health.

[20] Townsend C.L., Maynard R.L. (2002). Effects on Health of Prolonged Exposure to Low Concentrations of Carbon Monoxide. Occup. Environ. Med..

[21] Raub J.A., Mathieu-Nolf M., Hampson N.B., Thom S.R. (2000). Carbon Monoxide Poisoning—A Public Health Perspective. Toxicology.

[22] Cameletti M. (2020). The Effect of Corona Virus Lockdown on Air Pollution: Evidence from the City of Brescia in Lombardia Region (Italy). Atmos. Environ..

[23] Islam M.S., Rahman M., Tusher T.R., Roy S., Razi M.A. (2021). Assessing the Relationship between COVID-19, Air Quality, and Meteorological Variables: A Case Study of Dhaka City in Bangladesh. Aerosol Air Qual. Res..

[24] Petetin H., Bowdalo D., Soret A., Guevara M., Jorba O., Serradell K., Pérez García-Pando C. (2020). Meteorology-Normalized Impact of the COVID-19 Lockdown upon NO_2_ Pollution in Spain. Atmos. Chem. Phys..

[25] Tian H., Xu R., Canadell J.G., Thompson R.L., Winiwarter W., Suntharalingam P., Davidson E.A., Ciais P., Jackson R.B., Janssens-Maenhout G. (2020). A Comprehensive Quantification of Global Nitrous Oxide Sources and Sinks. Nature.

[26] Sutton M.A., Howard C.M., Erisman J.W., Billen G., Bleeker A., van Grinsven H., Grizzetti B. (2011). The European Nitrogen Assessment: Sources, Effects and Policy Perspectives.

[27] Grad R. (1999). Health Effects of Air Pollution. Lancet.

[28] Srimuruganandam B., Shiva Nagendra S.M. (2012). Source Characterization of PM 10 and PM 2.5 Mass Using a Chemical Mass Balance Model at Urban Roadside. Sci. Total Environ..

[29] Petrescu V., Ciudin R., Cioca L.I., Isarie C.L., Trif B., Nederita V. (2015). The Impact of Traffic Related Pollution on Air Quality In Sibiu. Environ. Eng. Manag. J..

[30] Misra C., Geller M.D., Shah P., Sioutas C., Solomon P.A. (2001). Development and Evaluation of a Continuous Coarse (PM 10–PM 25) Particle Monitor. J. Air Waste Manag. Assoc..

[31] Li N., Wang M., Bramble L.A., Schmitz D.A., Schauer J.J., Sioutas C., Harkema J.R., Nel A.E. (2009). The Adjuvant Effect of Ambient Particulate Matter Is Closely Reflected by the Particulate Oxidant Potential. Environ. Health Perspect..

[32] Arghya Sardar P.R. (2015). SO_2_ Emission Control and Finding a Way out to Produce Sulphuric Acid from Industrial SO_2_ Emission. J. Chem. Eng. Process Technol..

[33] Ding G., Thuy N. (2020). Carbon Management. Advances in Carbon Management Technologies.

[34] Reddy M.S., Venkataraman C. (2002). Inventory of Aerosol and Sulphur Dioxide Emissions from India. Part II—Biomass Combustion. Atmos. Environ..

[35] Fioletov V.E., McLinden C.A., Krotkov N., Li C., Joiner J., Theys N., Carn S., Moran M.D. (2016). A Global Catalogue of Large SO_2_ Sources and Emissions Derived from the Ozone Monitoring Instrument. Atmos. Chem. Phys..

[36] Shakil M.H., Munim Z.H., Tasnia M., Sarowar S. (2020). COVID-19 and the Environment: A Critical Review and Research Agenda. Sci. Total Environ..

[37] Eroğlu H. (2021). Effects of COVID-19 Outbreak on Environment and Renewable Energy Sector. Environ. Dev. Sustain..

[38] SanJuan-Reyes S., Gómez-Oliván L.M., Islas-Flores H. (2021). COVID-19 in the Environment. Chemosphere.

[39] Filonchyk M., Hurynovich V., Yan H. (2021). Impact of COVID-19 Pandemic on Air Pollution in Poland Based on Surface Measurements and Satellite Data. Aerosol Air Qual. Res..

[40] Balasubramaniam D., Kanmanipappa C., Shankarlal B., Saravanan M. (2020). Assessing the Impact of Lockdown in US, Italy and France—What Are the Changes in Air Quality?. Energy Sources Part A Recover. Util. Environ. Eff..

[41] Paital B. (2020). Nurture to Nature via COVID-19, a Self-Regenerating Environmental Strategy of Environment in Global Context. Sci. Total Environ..

[42] Berman J.D., Ebisu K. (2020). Changes in U.S. Air Pollution during the COVID-19 Pandemic. Sci. Total Environ..

[43] Muhammad S., Long X., Salman M. (2020). COVID-19 Pandemic and Environmental Pollution: A Blessing in Disguise?. Sci. Total Environ..

[44] Rupani P.F., Nilashi M., Abumalloh R.A., Asadi S., Samad S., Wang S. (2020). Coronavirus Pandemic (COVID-19) and Its Natural Environmental Impacts. Int. J. Environ. Sci. Technol..

[45] Jephcote C., Hansell A.L., Adams K., Gulliver J. (2021). Changes in Air Quality during COVID-19 ‘Lockdown’ in the United Kingdom. Environ. Pollut..

[46] Vultaggio M., Varrica D., Alaimo M.G. (2020). Impact on Air Quality of the COVID-19 Lockdown in the Urban Area of Palermo (Italy). Int. J. Environ. Res. Public Health.

[47] Tobías A., Carnerero C., Reche C., Massagué J., Via M., Minguillón M.C., Alastuey A., Querol X. (2020). Changes in Air Quality during the Lockdown in Barcelona (Spain) One Month into the SARS-CoV-2 Epidemic. Sci. Total Environ..

[48] Lokhandwala S., Gautam P. (2020). Indirect Impact of COVID-19 on Environment: A Brief Study in Indian Context. Environ. Res..

[49] Bao R., Zhang A. (2020). Does Lockdown Reduce Air Pollution? Evidence from 44 Cities in Northern China. Sci. Total Environ..

[50] Sharma S., Zhang M., Anshika, Gao J., Zhang H., Kota S.H. (2020). Effect of Restricted Emissions during COVID-19 on Air Quality in India. Sci. Total Environ..

[51] Mahato S., Pal S., Ghosh K.G. (2020). Effect of Lockdown amid COVID-19 Pandemic on Air Quality of the Megacity Delhi, India. Sci. Total Environ..

[52] Fiasca F., Minelli M., Maio D., Minelli M., Vergallo I., Necozione S., Mattei A. (2020). Associations between COVID-19 Incidence Rates and the Exposure to PM2.5 and NO_2_: A Nationwide Observational Study in Italy. Int. J. Environ. Res. Public Health.

[53] Díaz-Avalos C., Juan P., Chaudhuri S., Sáez M., Serra L. (2020). Association between the New COVID-19 Cases and Air Pollution with Meteorological Elements in Nine Counties of New York State. Int. J. Environ. Res. Public Health.

[54] Hutter H.P., Poteser M., Moshammer H., Lemmerer K., Mayer M., Weitensfelder L., Wallner P., Kundi M. (2020). Air Pollution Is Associated with COVID-19 Incidence and Mortality in Vienna, Austria. Int. J. Environ. Res. Public Health.

[55] Jasarevic T., Thomas G., Osseiran N. 7 Million Deaths Annually Linked to Air Pollution. https://www.who.int/news/item/25-03-2014-7-million-premature-deaths-annually-linked-to-air-pollution.

[56] Worldmeter Data. https://www.worldometers.info/coronavirus/coronavirus-death-rate/.

[57] Isaifan R.J. (2020). The Dramatic Impact of Coronavirus Outbreak on Air Quality: Has It Saved as Much as It Has Killed so Far?. Glob. J. Environ. Sci. Manag..

[58] González Ortiz A., Gsella A., Guerreiro C., Soares J., Horálek J. (2021). Health Risk Assessment of Air Pollution.

